# Spaying Mares as a Remedy for Vice

**Published:** 1901-10

**Authors:** A. H. Ide

**Affiliations:** Lowville, N. Y.


### REPORTS OF CASES.

### SPAYING MARES AS A REMEDY FOR VICE.

By A. H. Ide,
LOWVILLE, N. Y.

Sterilize instruments by boiling for five minutes in bicarbonate soda solution. Have in readiness three or four quarts of 1 : 1000 solution of corrosive sublimate. Fill irrigator with sterile bicarbonate soda solution, which is suspended above the operator's head. Thoroughly scrub the hips, tail, and vulva ; empty rectum ; hands thoroughly washed (special attention being given to finger-nails), and then rinsed in 1 : 1000 corrosive sublimate solution.

Irrigate the vagina with the sterile bicarbonate soda solution with a sterilized scalpel. Introduce the right hand into the vagina promptly, and when "the vagina is well bellowed" unsheath the knife, and, placing it just above the os uteri, push it directly forward in a straight line with a quick thrust through the vaginal mucosa, muscular walls, and the peritoneum until the disappearance of resistance indicates that the peritoneum has been penetrated. This is the most critical step in the operation.

Insert one finger in the incision, then a second, and, holding the fingers in the form of a cone, push the entire hand into the peritoneal cavity. With the palmar surface of the hand downward trace the horns of the uterus to the ovaries. Withdrawing the hand, convey the écraseur enclosed within the hand through the vaginal wound to the region of the ovary, release the écraseur, and retrace the parts if necessary, and, locating the ovary, drop the chain over the ovary from above, and either grasp the ovary with the fingers through the chain loop from above and draw it into the loop, or, passing one or two fingers around beneath the ovary, push it up through the loop to the grasp of the thumb and index finger above. Holding the ovary with one hand, tighten the chain quickly with the other; examine to make sure that a loop of the intestine is not caught; draw the ovary well through and get a large portion of the oviduct and cut off promptly, holding to the ovary until brought out through the vulva.

Bay mare, six years old. Vice: Switching tail and kicking in harness. Spayed under strict aseptic precaution. Recovery. Allowed to run in pasture for a period of two months, and, on being tested, was found kind and gentle.

Bay mare, aged seven years, and a black mare, aged six years. Vice: Switching tail and kicking in harness. Spayed at one sitting; aseptic precaution practically ignored. In about forty-eight hours from the time of the operation there were manifestations of local infection of the vaginal wound, marked by more or less intense colic, latter remittent or intermittent, almost constant straining, as in the act of voiding urine, accompanied by violent expulsive exertions. Vaginal palpation revealed the site of operation indurated and sensitive to pressure. Treatment consisted of packing the vaginal wound with absorbent cotton saturated with peroxide of hydrogen, followed by packing the vagina with a sheet of cloth saturated with creolin solution. Treatment repeated twice daily.

The bay mare commenced to improve at once, and after about eight days was turned out for a period of one month, and when tested in harness was found to be gentle, notwithstanding her very vicious traits before being operated upon. The other developed a case of septic peritonitis which resulted in death.

Black mare, aged eight years, used as a family driver, had shown almost constant heat for more than a year, would switch incessantly in harness, and when in the presence of other horses would become very much agitated and switch and urinate profusely. This mare was gentle to handle in the stable, and never showed a disposition to kick in harness. Spayed and caudal myectomy performed August 10, 1899. She recovered without evidence of inconvenience, and was driven in eight days from the date of the operation. In answer to a letter of inquiry bearing the date of September 10th, the owner reported the mare doing well and not showing any of her old traits, and gave it as his opinion that the operation would prove effective. About three weeks later I received a letter from the owner stating that the mare had taken up her old habit of switching. In answer I advised the owner to try Prof. Oscar R. Gleason's method of treating switchers, and enclosed full directions for its application. A later report reads: "We applied the Gleason treatment, and it did the work. The mare does not switch ; it is a pleasure to drive her now."

A still later report reads : "Have you still another remedy for switching. Your last treatment did nicely for a time, but is loosing its effect. If you could control that everlasting switching the other troubles would go with it." I advised the application of Gleason's treatment sufficiently often to keep control of the tail, which was sure of breaking up the habit. In a communication bearing date of July 31, 1901, the owner says that he had neglected to follow the Gleason treatment, and reported the mare in the same condition as before being operated upon.

I append the Gleason treatment, but do not intend to suggest that it can properly replace surgical treatment or spaying in cases where that is indicated. As we know of no essential bond of sympathy between the tail and genitals, we can scarcely assume that paralysis of the tail has effected a cure through the medium of the reproductive system. We are led to think it possible, if not probable, that the education given an animal by securing the tail fixedly over the sacrum for a period of six hours three days in succession (which constitutes the Gleason treatment), inflicting pain and rendering the parts powerless in a region about which it has previously been viciously irritable, plays an important part in the eradication of "vice." I can indorse this assumption, guided by practical demonstration on a goodly number of switching, kicking mares, and have found this method a very powerful and effective method of control.

Black mare, aged seven years. Vice : Kicking and uncontrollable in harness. The operation of spaying was undertaken in slings and carried to the first step, but had to be abandoned on account of the sling pulley breaking and letting the mare down. Further procedure was deferred for a period of ten days for the purpose of allowing the initial vaginal wound time to heal, at which time the mare was confined in stocks and the operation of spaying completed. The mare did nicely for a period of eight days, when she was reported not acting well and showing symptoms of colic. Vaginal examination revealed evidence of an abscess, which developed very fast. Treatment consisted of opening the abscess into the vagina by thrusting the index finger through the walls, followed by flushing the abscess cavity with antiseptics twice daily for ten days, which resulted in recovery. On being tested this mare proved to be a little cranky in harness, but has continued to do her work well, notwithstanding her uncontrollable disposition previous to operation.

Black mare, aged six years. Vice: Switching; had kicked once. Spayed. Means of restraint, stocks. This patient made a perfect recovery, and did not show at any time the least inconvenience from the operation. Test proved this mare perfect, which shows that "spaying" should be resorted to early, before the nervous system becomes too weakened.

Bay road mare; spayed at the Association last year, caudal myectomy by Dr. Fisher. In the October number of the *American Veterinary Review*, which gave the final outcome of the patients operated upon at the clinic, Prof. Williams writes: "The mare owned by Dr. Hoff exhibited no inappetence or inconvenience, and was driven home ninety miles in about a week after the meeting." Dr. Hoff writes: "The mare is all O. K. No more foolish actions, drives as nice as she can, does not squeal any more in the stable, nor urinate when I speak to her in the barn or the road. Am well pleased."

Black mare, aged nine years. Vice: Kicking. This mare was owned and driven by a lady, and was considered kind but not agreeable at times, as she had acquired the habit of switching. All went well until she gripped the line and ran away. The mare went into the hands of a second owner, who hitched her to a milk cart. She became uncontrollable and ran away a second time. Upon coming into the hands of a third party she was submitted for operation. Spayed, and caudal myectomy performed, July 1, 1901. I experienced some difficulty in operating on this patient. In tearing through the walls of the vagina the hand was carried too low and entered the interior of the uterus. A second incision, made about one inch superior to the superior border of the first incision, was effective, allowing the hand to pass with ease. Next day the mare ate well and acted bright. There was a slight degree of tympanism, which remained for a time. On the fourth day the mare refused her grain, but ate a little hay. Temperature, 103°; pulse, 56. Respiration quick and short, with double muscular action at the flank, which gave the impression that the patient was broken-winded. Treatment: Aloin, gr. iij, to act as a laxative; atropia, gr. $\frac{1}{4}$, twice daily; acetinilid, 2 drachms, every six hours, continued for two days. The atropia revealed the irregular breathing at once, and after the second day's treatment the patient commenced to improve rapidly, and was discharged July 8th. Placed in the team July 16th. Owner reports the mare kind, and says that she has not made an attempt to kick as yet.

Bay road mare. Vice: Switching, and generally disagreeable. Spayed, and caudal myectomy performed on August 5, 1901. This patient recovered without incident, and seemed to be in perfect health up to about ten days ago. The owner observed that the mare was not acting well, and said that she looked bloated. Prescribed a laxative and advised a light diet for a few days. Owner came in and reported the mare groaning worse; said she seemed to be in great pain, and was getting her hips raw from a persistent desire to press backward against some solid substance. I advised the owner to have the mare brought to my stable. Physical examination revealed the mare emaciated; vaginal palpation gave evidence of an abscess. Treatment: Opened the abscess by forcing the finger through the walls, which was followed by the discharge of about three quarts of pus. After treatment, consisting of flushing the abscess cavity with antiseptics twice daily, the patient is doing well at this writing, and her condition favorable to recovery.

Gray mare, aged ten years. Vice: Periodical kicking; constant switching in harness. Spayed, and superior inferior and lateral caudal myectomy performed, which consists in removing all the muscles from the tail, except the compressor coccygeus, the action of the tail being confined to the last-named muscle.

The patient did well, was discharged August 21st, and was allowed one week's rest, when she resumed her place in the team.

The owner reports the mare doing her work well, and not inclined to kick, and says what pleases him most she does not switch.

My observations and research prove conclusively to me that the spaying of mares is not merely a theoretical departure from set veterinary practice, but a practical acquisition to the profession.

---

*The Fourteenth Annual Report* of the Agricultural Experiment Station of Nebraska gives some interesting data showing analyses of a number of the swine-plague cures on the market.

